# Chagas disease: an impediment in achieving the Millennium Development Goals in Latin America

**DOI:** 10.1186/1472-698X-7-7

**Published:** 2007-08-28

**Authors:** Carlos Franco-Paredes, Anna Von, Alicia Hidron, Alfonso J Rodríguez-Morales, Ildefonso Tellez, Maribel Barragán, Danielle Jones, Cesar G Náquira, Jorge Mendez

**Affiliations:** 1 Hospital Infantil de México, Federico Gómez, México D.F; 2 Department of Medicine, Emory University School of Medicine, Atlanta GA, USA.^3 ^Centro Trujillano de Investigaciones Parasitológicas José Witremundo Torrealba, Universidad de Los Andes, Trujillo, Venezuela; 3 Institutos Nacionales de Salud de Perú, Lima Perú

## Abstract

**Background:**

Achieving sustainable economic and social growth through advances in health is crucial in Latin America within the framework of the United Nations Millennium Development Goals.

**Discussion:**

Health-related Millennium Development Goals need to incorporate a multidimensional approach addressing the specific epidemiologic profile for each region of the globe. In this regard, addressing the cycle of destitution and suffering associated with infection with *Trypanosoma cruzi*, the causal agent of Chagas disease of American trypanosomiasis, will play a key role to enable the most impoverished populations in Latin America the opportunity to achieve their full potential. Most cases of Chagas disease occur among forgotten populations because these diseases persist exclusively in the poorest and the most marginalized communities in Latin America.

**Summary:**

Addressing the cycle of destitution and suffering associated with *T. cruzi *infection will contribute to improve the health of the most impoverished populations in Latin America and will ultimately grant them with the opportunity to achieve their full economic potential.

## Background

### The Millennium Development Goals in Latin America

In 2000, the United Nations Millennium Summit adopted the Millennium Development Goals (MDGs) to address extreme poverty [1]. The purpose of these goals is multidimensional: promotion of education and environmental sustainability; alleviation of hunger; fostering gender equality; and improving the health of impoverished populations to encourage economic growth and reduce poverty and inequality [1,2]. In Latin America, these goals were reaffirmed in the Declaration of the Special Summit of the Americas, which was held in Monterrey, Mexico in 2004 [2]. Almost 3 years after this event, progress in poverty alleviation programs is insufficient to meet the goal of halving the proportion of people living in extreme poverty in the region by the year 2015 [2]. In order to achieve these goals, more than 9 million children need to attend school; many millions of women and girls need to enjoy enhanced participation in society and security. In addition, people need to drink safe water, enjoy basic sanitary conditions, allowing for healthier lives. All of these aspects should occur within the context of environmental sustainability [1,2].

Indeed, there are significant challenges ahead in order to make substantial strives with the existing living conditions and available resources in Latin America. In fact, achieving sustainable growth through advances in health is crucial in Latin America [3,4]. In addition, the frequently cited causes of poor health in the region [3], a group of diseases now defined as NTDs are responsible for a substantial deterioration of the health status of people living in extreme poverty [4]. They are named *neglected *because these diseases persist exclusively in the poorest and the most marginalized communities [3]. The diseases thrive in places with unsafe water, poor sanitation, and limited access to basic health care [4]. Despite the enormous disabilities they cause, these diseases are often less visible and given a low priority alongside high mortality diseases. Furthermore, the economic impact of NTDs can be staggering and thus, a never ending cycle of destitution ensues [4]. Therefore, it is critical to adapt the health-related MDGs to the epidemiologic profile of the region. In this regard, Chagas disease is a premiere example of the significant burden of disease (see next section) and suffering imposed by highly prevalent NTDs in the Americas.

### The unnecessary burden of Chagas disease

Chagas disease, also known as American trypanosomiasis, is a zoonotic tropical disease caused by the parasite *Trypanosoma cruzi *[5]. Millions of inhabitants of rural Mexico and Central and South America have, throughout history, been burdened by Chagas disease [5-7]. Not only does primary infection continue to endanger the lives of countless people in the region, but the chronic manifestations of Chagas disease also affect the livelihood of many individuals previously infected. As a result, Chagas disease remains the most important vector-borne neglected disease in the Americas today [2]. As the trend for global migration increases, the scope of Chagas disease threatens to expand exponentially, from rural to urban areas and endemic to non-endemic regions [7,8].

**Figure 1:**
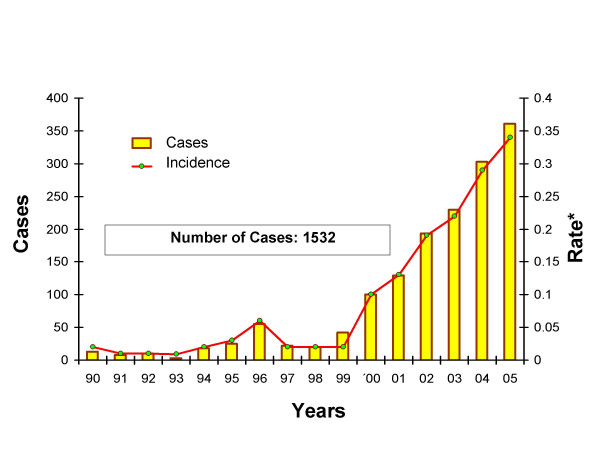
**Title Incidence of Chagas disease in Mexico, 1990–2005**. * Includes acute and chronic cases Source: Ministry of Health, Mexico CENEVECE (1990 – 2005) per 100 000 population

Despite significant decreases in infection and mortality that have been achieved through vector and transfusion-related control initiatives in Central and South America over the last decade, [5,7,8] current estimates suggest that ten to thirteen million people are chronically infected with *Trypanosoma cruzi *and approximately 90 million individuals remain at risk of contracting the infection [7,9]. In countries such as Mexico, increased surveillance combined with socioeconomic deterioration, especially in the Southern States, has led to increased incidence of Chagas (Figure 1) [10].

American trypanosomiasis manifests with acute and chronic (early and late) phases [5,7-9,11]. In some individuals, parasitization of cardiac muscle or brain during acute infection can lead to myocarditis or meningoencephalitis, respectively. If left untreated, the acute phase progresses to a subclinical latent period after several weeks (chronic early) and eventually may progress to a chronic late phase. Approximately 20 to 30% of those chronically infected are symptomatic [7-9]. For example, persistent *T. cruzi *infection can lead to unremitting inflammation in the myocardium, disrupting the cardiac conduction system and producing structural alterations. These events result in cardiac arrhythmias, congestive heart failure, thromboembolic events, and sudden cardiac death [5,7-9]. Destruction of visceral autonomic neurons in the digestive tract results in progressive dilation of the esophagus and colon and gastrointestinal dysmotility [5,11]. Unlike Chagas disease seen in immunocompetent patients, meningoencephalitis dominates the clinical picture in untreated HIV-infected patients [12].

As in all neglected diseases, Chagas disease creates financial and social burdens to the individual, his or her household and country, and continental Latin America [13-15]. Estimates suggest that the burden of Chagas disease in this region is, in fact, five to ten times larger than that of malaria [2]. The early mortality and substantial disability caused by this disease, which often occurs in the most productive population, young adults, results in a devastating economic loss in the Americas. In 1995, this was estimated at US$8,156 million, which is equivalent to 2.5% of the external debt of continental Latin America [14,15]. Older estimates suggest that $300 million dollars were lost in Brazil between 1979 and 1981 based on the total number of cases of Chagas disease in that country during that period of time [14,15]. More recent data demonstrate that globally, Chagas disease is associated with 14,000 deaths per year and 0.7 million disability-adjusted life-years, constituting the sixth most important neglected tropical disease worldwide [16].

## Discussion

### Poverty as the underlying determinant of Chagas disease

***" If it wasn't for typhus, malaria, Chagas disease, ignorance, and other highly successful plagues luckily spread out in rural areas, how would we extend the limits of our haciendas?, Without these plagues, how would we able to promote enough fear, and being able to continue to exploit the poor in the most effective way?***[17]"

Populations at risk of acquiring vector-borne, blood transfusion related, and congenital *T. cruzi *infection are often bound more by socioeconomic status than by latitude [15]. The most impoverished populations live in poor quality housing with substandard conditions across Latin America that constitute a prime habitat for *T. cruzi *vectors [1,10-12,16]. Unsafe blood-transfusion practices and inadequate access to adequate prenatal care offers the opportunity for Chagas disease to ravage underserved, vulnerable populations [11,15].

**Figure 2:**
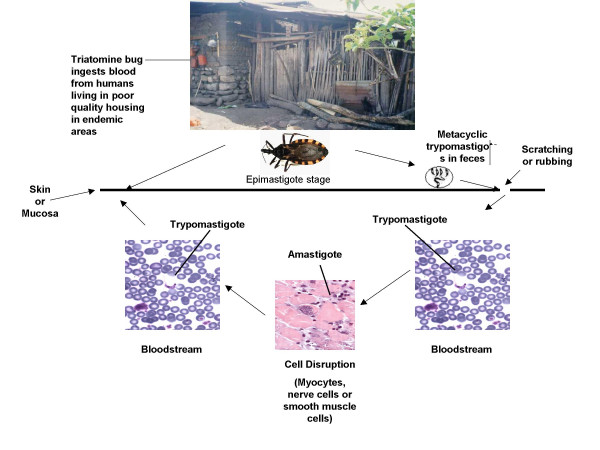
**Title Cycle of transmission of *Trypanosoma cruzi *and its vectors to humans**. Triatomine bugs live in the crevices of poorly constructed houses in impoverished areas in Latin America. Metacyclic trypomastigote is the infecting form to humans, while the amastigote is the intracellular form responsible for the immunopathogenesis in target human organs.

Vector-borne cases, the most important form of acquisition of the disease, result from the pervasive presence of transmitting vectors in poorly constructed human dwellings [5,11,14,18,19]. Transmission arises when insects of the *Reduviidae *family, subfamily *Triatominae *bite humans or other mammals. While the triatome ingests a blood meal, inoculation occurs as *T. cruzi *is excreted via feces into breaks in the skin or mucosa of the victim (Figure 2) [5,7,11]. Although a large number of species exist, the most important vectors to humans are *Triatoma infestans*, *Rhodnius prolixus*, *Triatoma dimidiata*, and *Panstrongylus megistus *[7,15,18,19]. The triatomes' wild habitat typically includes palm trees, tree holes, burrows, rock crevices, or other animal refuges, but some of these insects have learned to adapt to live and thrive in human dwellings. During the day, triatomines may hide in dark crevices or in unplastered, cracked walls of mud or mud-brick houses [18,19]. In order to meet their basic needs and ensure their immediate survival, many individuals are forced to accept the long-term consequences of sharing their household with these vectors. In many Latin American cities where Chagas disease is endemic, unregulated urbanization and widespread emergence of shantytowns has created serious strain on community resources and left many neighborhoods without a basic infrastructure, further exacerbating the cycle of destitution associated with this disease [3-5,11,19]. Furthermore, triatomines living in the peridomestic and sylvatic environments can also be transmitted by contaminated food with the consequent oral transmission resulting in microepidemics of acute cases of Chagas disease [20].

Poor housing, lack of access to safe blood-bank supplies, and inadequate prenatal care are linked to social and health inequalities that prevail in many areas of Latin America [5,7,15-18,21]. Such related social ills occur among the most impoverished Latin American populations and their roots are entrenched within large-scale social structures – political and economical [4]. Many people forgotten by their governments are coincidentally, the same people who encounter infection with *T. cruzi*. The vast majority with Chagas has the lowest incomes, poor sanitation and nutrition, the worst opportunities for education, low quality housing, and endure an inter-generational persistence of social inequalities [3,4]. Poverty not only restricts patients' access to diagnosis and treatment of the disease [4,11,16], but it leads to malnutrition, thereby limiting an individual's ability to recover and return to work. In a vicious cycle, poor living conditions lead to increased incidence of Chagas disease which cripples the population leaving it unable to work, gain revenue, and reach its full potential [4]. Poverty and underdevelopment, therefore, persist.

Many of the ill have also been exploited and deprived of basic human rights by either members of the wealthy social classes or their governments [4]. Some marginalized groups, feeling that there is no alternative to their survival, manifest their misery and hunger for social equity with uprisings against the inequalities that have reigned their lives for decades [4,17]. An illustration of these upheavals occurred in response to the growing health and social inequalities prevailing in Chiapas Mexico in 1994. Pleading for justice, subcomandante Marcos wrote a letter to the Mexican government which declared: *"Should we ask pardon from the dead, those who die of 'natural' deaths of 'natural causes' like measles, whooping cough, Chagas disease, cholera, typhoid, tetanus, pneumonia, gastrointestinal infections, and malaria?..... Our dead, the democratically dead, dying from sorrow because no one did anything, because the dead, our dead, went just like that, without anyone saying enough."*

### Chagas disease and the Millennium Development Goals

Chagas disease and other neglected diseases separate those living in endemic areas from the moral premise of the Universal Declaration of Human Rights and far from contributing to the achievement the MDGs [1,2,4]. Untreated Chagas disease impairs human development in this region by producing substantial morbidity, premature death, and disability that limits the ability of both current and future generations to meet their basic human requirements including sufficient income [1,2,4]. Additionally, the direct and indirect costs of *T. cruzi *infection impose an overwhelming load to the healthcare system secondary to hospitalizations and medical and surgical treatments for chagasic cardiomyopathy, gastrointestinal dysfunction, and meningoencephalitis [14,15]. Of course, this only applies to those individuals that can afford or who have access to healthcare.

In order to reach the MDGs in Latin America by 2015, approximately 118 million people will have to be lifted out of extreme poverty [2]. Public health policies and programs directed at controlling neglected tropical diseases in Latin America, in particular, Chagas disease must occur concomitantly with policies to accelerate investment, reduce poverty, and promote social development [1,4]. In this regard, control of Chagas disease requires that each mode of transmission be addressed. The main challenges in the new millennium, therefore, include vector control programs, housing improvement programs, comprehensive and easily accessible medical care, continuation and expansion of blood-screening programs, and treatment of currently infected individuals.

### Vector control programs

There have been successes in the battle against Chagas disease using household insecticides and blood-bank screening through the Southern Cone Initiative [14,15,18-20] Continued vector control activities with consolidation of operational control programs and continued political commitment with strong leadership and synchronization of efforts among the programs managed through the different Latin American governments is required for sustainable success [3,4]. Impregnated mosquito nets may also be useful for self-protection, particularly in areas with geographic overlap with malaria [16]. Despite some improvements in the control of this infection and the importance of vector control programs, *T. cruzi *infects several insect species and has multiple animal reservoirs. Successful elimination of one species, as it has occurred in domestic Chagas disease transmission in Brazil and other Southern Cone Initiative countries, may lead to the occupation of vacant niches by other triatomines species. As an example some the golden lion tamarins in the Poço das Antas National Park (only 60 km from Rio de Janeiro) have been recently found to be infected with the type II lineage of *T. cruzi*. In this manner, sylvatic species may adapt to an anthropomotic transmission, and reinvasion of insects from sylvatic transmission cycles becomes a major challenge for Chagas disease control [19,20].

Growing efforts to prevent further transmission through blood transfusion and organ donation screening programs are also fundamental [9-11,18,21].

### Housing improvement programs

Combining vector control practices with housing improvement initiatives will concomitantly target many MDGs [12,15,20]. By modifying houses and their immediate surroundings, long-term protection from triatomine bugs can be achieved. Filling cracks in walls and roofs with plaster prepared from locally available materials can decrease the number of resting places for the insects in a cost-effective manner [10,11]. Additionally, coating house-frames, walls and roofs with insecticidal paints in endemic areas has shown some benefit [3,10,11]. The addition of educational campaigns to modify storage places for agricultural products, firewood or other belongings to current campaigns such as those existing for dengue fever, may promote the reduction of habitats for the vectors [7]. Fortunately, these improvements are relatively economical when compared with the cost of chronic Chagas disease. It has been estimated that the cost to improve or construct more than 700,000 dwellings in rural Brazil is equal to US$750 million, the same amount spent annually in Brazil for medical and surgical management of chronic Chagas disease [14,15].

### Improved case management

Control of Chagas disease requires adequate treatment of chronic cases of the disease. Currently, there is no adequate treatment for chronic late cases, which are associated with most of the morbidity and mortality of Chagas disease [5,8,11]. Nifurtimox and benznidazole are effective only in the treatment of acute and chronic early phase cases and work primarily by preventing the occurrence of late chronic disease [8,11]. Expanding knowledge of parasite metabolism has renewed hope for effective chemotherapy for chronic phase *T. cruzi *infection [8,11]. Drug targets consist of recently identified critical metabolic pathways in the life cycle of this parasite, including blockage of sterol synthesis, cysteine protease metabolism, farnesyl pyrophosphate and others. Parasitologic elimination using the above strategies may provide an opportunity to prevent the onset of late manifestations when used early and even halt the progression of the disease when used late [8,11]. In addition, efforts to control Chagas disease, modeled after the rapid-impact programs to alleviate the burden of tropical neglected diseases in sub-Saharan Africa [16], could have a rapid and visible payoff on the health status of millions in Latin America.

## Summary

We should take advantage of the growing momentum in the fight against neglected tropical diseases. In this regard, a new effort to eliminate Chagas disease by 2010 has been recently launched by WHO through the establishment of a Global network to combat the disease. This collaborative initiative involves WHO Headquarters, the PAHO, non-governmental organizations, academic centers, and pharmaceutical industry [22]. Addressing the cycle of destitution and suffering associated with *T. cruzi *infection will ultimately grant the most impoverished populations in Latin America the opportunity to achieve their full potential. American trypanosomiasis can cease to be the shameful shadow that defines poverty and underdevelopment in Latin America [5].

## Abbreviations

MDGs, Millennium Development Goals; NTDs, Neglected Tropical Diseases; WHO, World Health Organization; PAHO, Pan-American Health Organization

## Competing interests

The author(s) declare that they have no competing interests.

## Authors' contributions

**CFP **conceived the idea of this work and contributed to the writing of the manuscript. **AV **conceived the idea of this work and contributed to the writing of the manuscript. **AJRM **conceived the idea of this work and contributed to the writing of the manuscript. **IT **conceived the idea of this work and contributed to the writing of the manuscript. **AH **conceived the idea of this work and contributed to the writing of the manuscript. **DJ **participated in the review of the literature and to the writing of the manuscript and also in the revised version of the manuscript. **MB **participated in the review of the literature and to the writing of the manuscript and also in the revised version of the manuscript. **CGN **participated in the writing of the manuscript and senior experts in the topic of vector-borne diseases in Latin America. **JM **participated in the writing of the manuscript and senior experts in the topic of vector-borne diseases in Latin America All authors read and approved the final manuscript.

## Pre-publication history

The pre-publication history for this paper can be accessed here:



## References

[B1] Sachs JD, McArthur JW (2005). The Millennium Project: a plan for meeting the Millennium Development Goals. Lancet.

[B2] Inter-American Development Bank (2005). The Millennium Development Goals in Latin America and the Caribbean: Progress, priorities and IDB support for their implementation.

[B3] Riley LW, Ko AI, Unger A, Reis MG (2007). Slum health: diseases of neglected populations. BMC International Health and Human Rights.

[B4] Franco-Paredes C, Jones D, Rodriguez-Morales AJ, Santos-Preciado JI (2007). Improving the health of neglected populations in Latin America. BMC Public Health.

[B5] Pinto Dias JC (2006). The treatment of Chagas disease (South American trypanosomiasis). Ann Intern Med.

[B6] Aufderheide AC, Salo W, Madden M, Streitz J, Buikstra J, Guhl F, Arriaza B, Renier C, Wittmers LE, Fornaciari G, Allison M (2004). A 9,000-year record of Chagas disease. Proc Natl Acad Sci.

[B7] World Health Organization Chagas disease information. The UNICEF-UNDP- Programme on TDR.

[B8] The Lancet (2006). Chagas disease – an epidemic that can no longer be ignored. Lancet.

[B9] Maguire JH (2006). Chagas disease-Can we stop the deaths?. N Engl J Med.

[B10] Attaran A (2006). Chagas' disease in Mexico. Lancet.

[B11] Urbina JA, Docampo R (2003). Specific chemotherapy of Chagas disease: controversies and advances. Trends Parasitol.

[B12] CDC/NIH/ATS (2005). Treating opportunistic infections among HIV-infected adults and adolescents. MMWR.

[B13] Schofeld CJ, Dias JC (1999). The southern cone initiative against Chagas disease. Adv Parasitol.

[B14] Moncayo A (1997). Progress towards the elimination of transmission of Chagas disease in Latin America. World Health Stat Q.

[B15] Moncayo A (2003). Chagas Disease: Current epidemiological trends after the interruption of vectorial and transfusional transmission in the southern cone countries.

[B16] Hotez P, Molyneux DH, Fenwick A, Ottessen E, Ehrlich Sachs S, Sachs JD (2006). Incorporating a rapid-impact package for neglected tropical diseases with programs for HIV/AIDS, tuberculosis, and malaria. PLoS Med.

[B17] Amado Jorge (1987). Tereza Batista cansada de guerra.

[B18] Miles MA, Feliciangeli MD, Rojas de Arias A (2003). American trypanosomiasis (Chagas disease) and the role of molecular epidemiology in guiding control strategies. Brit Med J.

[B19] Cecere MC, Vazquez-Prokopec GM, Gurtler RE, Kitron U (2004). Spatio-tempral analysis of reinfestation by *Triatoma infestans *(Hemiptera: Reduviidae) following insecticide spraying in a rural community in Northwestern Argentina. Am J Trop Med Hyg.

[B20] De Souza W (2007). Chagas' disease: facts and reality. Microbes Infect.

[B21] Schmunis GA, Cruz JR (2005). Safety of blood supply in Latin America. Clin Microb Rev.

[B22] World Health Organization New global effort to eliminate Chagas disease. http://www.who.int/mediacentre/news/releases/2007/pr36/en/index.html.

